# Sublingual immunization with E2-CD154 protein and the STING agonist c-di-AMP confers protection against classical swine fever virus in pigs

**DOI:** 10.3389/fcimb.2025.1713724

**Published:** 2025-11-20

**Authors:** Sara Puente-Marin, Talía Sardina-González, Liani Coronado, Cristina Riquelme, Saray Heredia, Adriana Muñoz-Aguilera, Yusmel Sordo-Puga, Danny Pérez-Pérez, Alina Rodríguez-Mallon, Mario Pablo Estrada, Carlos A. Duarte, María Pilar Rodríguez-Moltó, Llilianne Ganges

**Affiliations:** 1 IRTA, Programa de Sanitat Animal, Centre de Recerca en Sanitat Animal (CReSA), Barcelona, Spain; 2 Unitat mixta d’Investigació IRTA-UAB en Sanitat Animal, Centre de Recerca en Sanitat Animal (CReSA), Barcelona, Spain; 3 WOAH Reference Laboratory for Classical Swine Fever, Institute of Agrifood Research and Technology, Centre de Recerça en Sanitat Animal (CReSA), Barcelona, Spain; 4 Departamento de Salud Animal, Centro de Ingeniería Genética y Biotecnología, La Habana, Cuba; 5 Subgerencia de Análisis y Diagnóstico, Instituto Colombiano Agropecuario (ICA), Bogotá, Colombia

**Keywords:** sublingual vaccination, c-di-AMP, recombinant subunit vaccine, classical swine fever virus (CSFV), DIVA strategy, porcine immunization, E2-CD154 chimeric protein

## Abstract

**Introduction:**

Subunit vaccines represent a safer alternative to live attenuated formulations. However, they often require potent adjuvants and delivery systems to elicit robust immunity, particularly against highly contagious diseases such as Classical Swine Fever (CSF).

**Methods:**

In this study, we investigated the immunogenicity and protective efficacy of a novel mucosal subunit vaccine comprising the chimeric E2-CD154 protein, co-administered with the mucosal adjuvant c-di-AMP, in domestic pigs. Optimal dosing and immunization schedules for sublingual immunization were determined, followed by a challenge experiment using a highly virulent CSF virus (CSFV) strain.

**Results:**

Our results showed that sublingual co-administration of E2-CD154 and the STING agonist c-di-AMP conferred robust clinical protection, effectively prevented viral replication, and restricted the dissemination of infectious virus. This combination induced strong systemic IgG and IgA responses and neutralizing antibodies against multiple CSFV strains, achieving outcomes comparable with the commercial Porvac^®^ vaccine, administered intramuscularly. Importantly, virus isolation from tonsils confirmed the absence of infectious virus in pigs immunized with E2-CD154 and c-di-AMP, unlike those receiving E2-CD154 or the adjuvant alone. Moreover, immunized animals exhibited minimal IFN-α serum levels post-challenge, indicating reduced innate activation and viral replication.

**Discussion:**

These findings provide evidence, in a large mammalian host such as the pig, that c-di-AMP functions as an adjuvant for a recombinant E2-CD154 protein delivered sublingually, enhancing immune responses consistent with protection against viral replication. Together, these results offer insights into the development of non-replicating, DIVA-compatible platforms against CSFV and support the rational design of next-generation subunit vaccines targeting viral pathogens relevant to both veterinary and human medicine.

## 1 introduction

Vaccination is one of the best public health approaches to protect and prevent against contagious diseases in a simple, safe, and effective way (The World Health Organization (WHO), 2025). Some infectious diseases still lack a vaccine, underscoring the need for further research to advance our understanding of disease pathogenesis and immunology, as well as to develop new vaccination strategies. Aspects such as the type of vaccine, dose, schedule, and route of administration, as well as its formulation, including immune adjuvants, are a cornerstone to improve vaccinology.

The subunit vaccines are based on specific antigens of a pathogen and are becoming the most popular design for modern vaccines. They are safe, stable, and easy to store, directing immune responses toward key antigenic targets (Rebecca et al., 2022). Despite these advantages, subunit formulations also present important limitations, as most require parenteral administration and multiple doses to achieve effective protection. Moreover, these vaccines elicit limited mucosal immunity and lack endogenous danger signals (pathogen-associated molecular patterns (PAMPs)), which activate innate immune pathways, which are essential for initiating robust adaptive responses. Although they are effective when administered parenterally, achieving successful oral delivery remains a major challenge. Enhancing mucosal immunogenicity is therefore a key objective, as oral vaccination could minimize adverse effects, improve patient compliance, and facilitate large-scale immunization in both humans and animals. Consequently, innovative formulations and adjuvant systems are urgently needed to potentiate mucosal immunity while retaining the safety profile, diagnostic compatibility, and broad applicability of subunit vaccines (Zhang et al., 2025).

To overcome this, subunit vaccines are almost always combined with adjuvants that mimic or enhance danger signals (Moyle, 2017; Thakur and Bailey, 2019). Cyclic di-adenosine monophosphate (c-di-AMP) is a recently discovered PAMP, first identified in *Bacillus subtilis* as a second messenger (Witte et al., 2008), which activates the stimulator of interferon genes (STING) activating host innate immune responses. In addition, c-di-AMP elicited a mucosal immune response in the host when used as an adjuvant (Cheng et al., 2022; Coderch et al., 2023), highlighting its potential as an immunostimulatory agent of recombinant vaccines. Experiments in mice have shown that c-di-AMP promoted local, effective cellular and systemic humoral responses, and protection against a wide range of pathogens including bacteria and some viruses (Sanchez et al., 2014; Ebensen et al., 2017; Landi et al., 2017; Schulze et al., 2017a; Schulze et al., 2017b; Muñoz González et al., 2019; Ning et al., 2021).

The sublingual route represents an excellent model for evaluating mucosal vaccine prototypes, providing direct access to the mucosal immune system while avoiding antigen degradation in the gastrointestinal tract. Although oral delivery remains the goal for veterinary applications, sublingual immunization offers a practical and reliable approach for optimizing formulations and adjuvants, such as c-di-AMP prior to developing oral mucosal vaccines. This route combines superior permeability and prolonged antigen retention compared with the buccal mucosa, resulting in stronger and more consistent immune responses (Kraan et al., 2014; Trincado et al., 2021). It also ensures efficient antigen uptake through a thin, highly vascularized mucosa enriched in antigen-presenting cells and has proven to elicit immune responses comparable with intranasal vaccination with c-di-AMP, but without associated safety concerns (Ebensen et al., 2017). Moreover, being needle-free, simple to administer, and well established in humans as sublingual immunotherapy, it represents a safe and promising platform for advancing mucosal vaccination strategies (Agostinis et al., 2005).

Herein, we focus on classical swine fever (CSF), a devastating hemorrhagic disease that continues to threaten the swine industry worldwide. The causative agent, classical swine fever virus (CSFV), is an enveloped RNA virus belonging to the genus Pestivirus. CSF is a notifiable disease to the World Organisation for Animal Health (WOAH), which recommends the use of marker vaccines capable of differentiating infected from vaccinated animals (DIVA concept). Such an approach has the potential to mitigate the negative impact of CSF on international trade and animal welfare. The E2-CD154 chimeric recombinant protein, consisting of the CSFV E2 glycoprotein fused to the porcine CD154 molecule, constitutes the active component of the Porvac^®^ subunit vaccine (Sordo-Puga et al., 2021). Administered parenterally, Porvac^®^ confers solid protection against CSFV and has proven effective in controlling field outbreaks (Suárez et al., 2017; Sordo-Puga et al., 2021; Sordo-Puga et al., 2025a; Sordo-Puga et al., 2025b). Owing to its DIVA compatibility, since it contains exclusively the immunogenic E2 glycoprotein, vaccinated animals remain seronegative for the CSFV E^rns^ glycoprotein, thereby enabling reliable serological differentiation from naturally infected pigs (Ganges et al., 2020). Moreover, the Porvac^®^ vaccine has demonstrated effective protection against CSFV transplacental transmission following a two-dose regimen (Muñoz-González et al., 2017). The wild pigs and boars are the main reservoirs of CSFV, and eradication programs focus on controlling the circulation of the virus in wild animals. In this scenario, effective oral immunization could constitute a key tool to enhance herd immunity and interrupt the chain of infection (Rossi et al., 2015; Hayama et al., 2023). However, to date, no subunit vaccine has been able to confer protection against CSF when administered orally.

Building on these precedents, the aim of this study was to conduct a proof-of-concept evaluation of c-di-AMP as a mucosal adjuvant for the E2-CD154 chimeric recombinant protein in domestic pigs. Specifically, we investigated the feasibility of sublingual delivery and its potential to enhance immunogenicity and confer protective responses following immunization and subsequent CSFV challenge under controlled experimental conditions. After immunization and CSFV challenge, the immunogenicity and efficacy were determined using the clinical score parameters and molecular and immunological tests. This proof-of-concept work represents an initial step toward next-generation, non-injectable subunit vaccine platforms.

## 2 Materials and methods

### 2.1 Cells and viruses

Viral production, titration, and neutralization assays were performed using porcine kidney cell line PK-15 (ATCC-CCL-33). The swine kidney cell line SK-6 was used for viral isolation using the peroxidase-linked assay (PLA) (Kasza et al., 1972; Wensvoort et al., 1986). The cell lines were grown in Eagle’s minimum essential medium supplemented with 5% foetal bovine serum. Cells were cultured in a 37°C incubator with a 5% CO_2_ atmosphere.

The CSFV strain Margarita (genotype 1.4) was used in the *in vivo* experiments and for virus neutralization assays. The CSFV Alfort/187 (genotype 1.1) and Diepholz1/Han94 (genotype 2.3) strains were kindly provided by the CSFV EU Reference Laboratory (EURL), Hanover, Germany, and used for virus neutralization assays.

### 2.2 E2-CD154 protein, Porvac^®^ vaccine, and c-di-AMP

The fusion of the extracellular region of E2 glycoprotein of CSFV Margarita strain and the extracellular segment of the swine CD154 molecule constitutes the chimeric protein E2-CD154. It was obtained from the stably transformed cell line HEK 293, as described elsewhere (Suárez et al., 2017). Briefly, cells were grown in a 10-L Biostat B Plus fermenter (Germany), with serum-free medium. The metabolized culture medium was concentrated in a tangential flow concentrator (PESU 100-kDa cassette, Sartorius, Germany) and filtered through a 0.2-µM membrane. The proteins used in all experiments corresponded to production batches 13P.2204, 13P.2301, and 13P.2318 of the Pharmaceutically Active Ingredient of Porvac^®^ vaccine, previously released by the Quality Control Department of the Center for Genetic Engineering and Biotechnology of Camagüey (CIGB-Camagüey, Cuba). The protein was stored at 4 °C until use. The E2-CD154 protein formulated in Montanide TM ISA50 V2 (SEPPIC, La Garenne-Colombes, France) in an aqueous/oil phase constitutes the commercially available Porvac^®^ vaccine (Sordo-Puga et al., 2021). c-di-AMP was purchased from IBIAN Technologies (Zaragoza, Spain).

### 2.3 Dose, inoculation route, and schedule immunization of E2-CD154 co-administered with c-di-AMP in pigs

**Figure 1:**
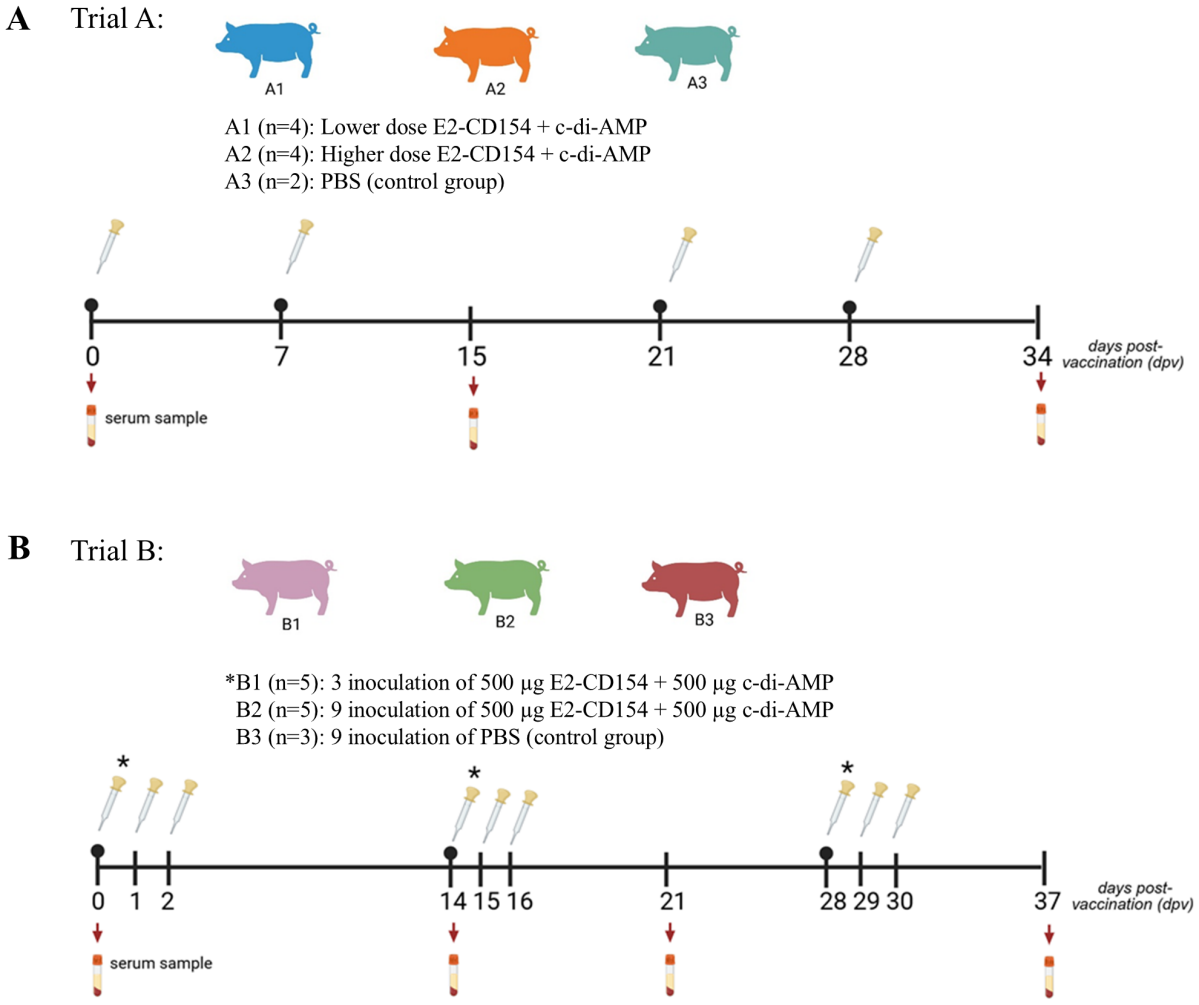
Experimental design of the dose and schedule immunization with E2-CD154 and c-di-AMP in pigs. **(A)** Experiment of dose concentration: Group A1, lower dose: 100 µg of E2-CD154 with 50 µg of c-di-AMP on days 0 and 7 and 100 µg of E2-CD154 with 200 µg of c-di-AMP on days 21 and 28. Group A2, higher dose: 500 µg of E2-CD154 with 50 µg of c-di-AMP on days 0 and 7 and 500 µg of E2-CD154 with 400 µg of c-di-AMP on days 21 and 28. Group A3, negative control, four doses of PBS. Blood samples were collected at 0, 15, and 34 dpv. **(B)** Experiment of schedule immunizations: Group B1, three inoculations of 500 µg of E2-CD154 with 500 µg of c-di-AMP on 0, 14, and 28 dpv. Group B2, nine inoculations of 500 µg of E2-CD154 with 500 µg of c-di-AMP on 0, 1, 2, 14, 15, 16, 28, 29, and 30 dpv. Group B3, negative control, nine inoculations of PBS on days 0, 1, 2, 14, 15, 16, 28, 29, and 30 dpv. Blood samples were collected at 0, 14, 21, and 37 dpv. Dpv, days post-vaccination. The red drops indicate blood extraction and serological pipettes the inoculation time.

The antibody response triggered by different concentration doses of the chimeric E2-CD154 protein in combination with c-di-AMP was evaluated in domestic pigs after sublingual administration (Trial A). A total of 10 Pestivirus-free pigs, approximately 10 kg in weight, were purchased from the National Center for the Production of Laboratory Animals (CENPALAB, Mayabeque, Cuba). Animals were randomly distributed into three groups. The group A1 (n=4), lower dose, was immunized with two doses on days 0 and 7, respectively, with 100 µg of E2-CD154 + 50 µg of c-di-AMP in phosphate-buffered saline (PBS: 50 mM phosphate buffer + 0.3 M NaCl, pH 7.2) per dose. On days 21 and 28, pigs received two more doses containing 100 µg of E2-CD154 + 200 µg of c-di-AMP per dose in PBS. The group A2 (n=4), higher dose, was immunized with two initial doses on days 0 and 7, respectively, with 500 µg of E2-CD154 + 50 µg of c-di-AMP. On days 21 and 28, this group received two more doses, with 500 µg of E2-CD154 + 400 µg of c-di-AMP per dose. The group A3 (n=2), negative control group, received only PBS at days 0, 7, 21, and 28. Serum samples were collected on days 0, 15, and 34 post-immunization (**Figure 1A**). For sublingual administration, the doses were placed directly under the tongue for 1 min. Pigs were deprived of water and food for 40 min before and after each inoculation. For the immunization schedule optimization (trial B), 13 Pestivirus-free domestic pigs, approximately 10 kg in weight, were purchased from CENPALAB. Animals were randomly assigned into three groups and immunized by sublingual administration as explained above. In group B1 (n=5), animals received three immunizations on days 0, 14, and 28 using 500 µg of E2-CD154 + 500 µg of c-di-AMP in PBS per dose. In group B2 (n=5), pigs were immunized with nine inoculations on days 0, 1, 2, 14, 15, 16, 28, 29, and 30, using 500 µg of E2-CD154 + 500 µg of c-di-AMP in PBS per dose. In group B3 (n=3), the animals were administered with PBS in a similar schedule to group B2. Animals were sampling on days 0, 14, 21, and 37 for sera collection (**Figure 1B**).

### 2.4 Experimental design to evaluate the immunogenicity and efficacy of E2-CD154 co-administered with c-di-AMP against CSFV

A total of 20 Pestivirus-free domestic pigs of 5 weeks of age were housed in the Animal Biosafety Level 3 (BSL3) facility at IRTA-CReSA (Barcelona, Spain). The animals were randomly allocated into four groups, and all handling procedures were performed in a blind manner for all groups. In accordance with previous studies, five animals per group were used (Sordo-Puga et al., 2021; Bohórquez et al., 2022; Sordo-Puga et al., 2025a).

**Figure 2:**
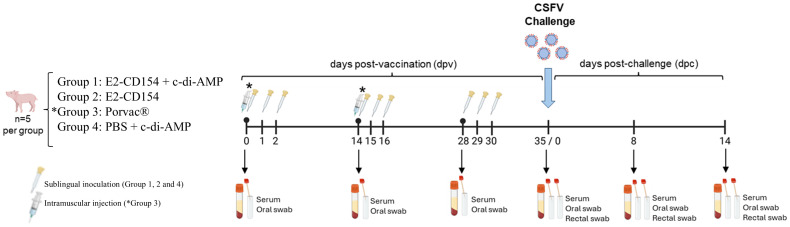
Experimental design to evaluate the immunogenicity and efficacy of E2-CD154 co-administered with c-di-AMP against CSFV. Group 1, nine sublingual inoculations of 500 µg of E2-CD154 with 500 µg of c-di-AMP on 0, 1, 2, 14, 15, 16, 28, 29, and 30 dpv. Groups 2, nine sublingual inoculations of 500 µg of E2-CD154 in the same schedule immunization than group 1. Group 3, two intramuscular injections of the standard dose of Porvac^®^ vaccine and 0 and 14 dpv, as positive control. Group 4, nine sublingual inoculations of PBS and 500 µg of c-di-AMP in the same schedule immunization than group 1 and 2. At 35 dpv, pigs were challenged with 10^4^ TCID_50_/mL of the highly virulent CSFV Margarita strain via oral. Serum and oral swab samples were collected at 0, 14, 28, and 35 dpv. Serum, oral, and rectal swab samples were collected at 8 and 14 dpc. Five pigs were used per group. Serological pipettes indicate sublingual inoculation in group 1, 2 and 4. Syringes indicate intramuscular injection in group 3. dpv, days post-vaccination. dpc, days post-challenge.

After 7 days of the acclimation period, group 1 was immunized with 500 µg of E2 CD154 + 500 µg of c-di-AMP and the group 2 with 500 µg of E2-CD154 alone per dose, both in a final volume of 2 mL and administered via sublingual, as described above. The doses were administrated at days 0, 1, 2, 14, 15, 16, 28, 29, and 30 (nine immunizations in total). Group 3 was vaccinated with the commercial Porvac^®^ vaccine via intramuscular, in two doses of 2 mL on day 0 and 14, as recommended by the manufacturer (Sordo-Puga et al., 2025a). Group 4 was administered with 500 µg of c-di-AMP in PBS via sublingual in a similar schedule and manner to groups 1 and 2 (**Figure 2**). At 35 days post vaccination (dpv), all groups were orally challenged with 10^4^ TCID_50_/mL of the highly virulent CSFV Margarita strain. After the CSFV challenge, a trained veterinarian recorded clinical signs daily, following previously described methodologies (Muñoz-González et al., 2017; Bohórquez et al., 2022). All sample collection and clinical monitoring were performed in a blinded manner throughout the trial. Sera and oral swab samples were collected at 0, 14, and 28 dpv. After CSFV challenge, sera, oral, and rectal swabs were collected at 0, 8, and 14 days post-challenge (dpc). At the end of the study (14 dpc), euthanasia of the animals was carried out following prior sedation using an intramuscular combination of 3 mg/kg xylazine and 15 mg/kg ketamine. The euthanasia was subsequently completed through an overdose of sodium pentobarbital at a dose of 90 mg/kg administered via the jugular vein in accordance with European Directive 2010/63/EU. After euthanasia, necropsies were carried out to confirm or discard the presence of CSFV-compatible lesions (data not shown) and tissue samples from tonsils were collected.

### 2.5 Ethics statement

Trials A and B protocols were approved by the Institutional Committee for the Use and Care of Laboratory Animals (CICUAL) of CIGB, under animal experimentation project numbers ECVVO0122 (20/04/2022) and ECVVO123 (05/05/2023). The experiments were in accordance with the international standards for animal welfare, following the regulations of the Cuban Ministry of Agriculture (MINAG).

The efficacy experiment against CSFV was approved by the Ethics Committee from the Government of Catalonia under animal experimentation project number 12497, 09/10/2024, in accordance with Spanish and European regulations. These ethic committees align with the ARRIVE guidelines.

### 2.6 Detection of antibody response against CSFV E2 and E^rns^ glycoproteins by ELISA

Three commercial kits were used to detect antibodies against CSFV E2 and E^rns^ glycoproteins. The PrioCHECK™ Porcine CSFV Ab 2.0 Strip Kit (Thermo Fisher Scientific Inc, Waltham, USA) was used to detect antibodies against E2 glycoprotein in the dose and immunization schedule optimization study (trials A and B), according to the manufacturer’s instructions. The optical density (OD) was measured at 450 nm. The OD_450_ of all samples is expressed as percent inhibition (PI). The positivity criterion for sera was a PI equal to or higher than 40%.

For the efficacy and immunogenicity study, E2-specific antibodies were detected in serum samples from all groups at 0, 14, and 28 dpv and at 0, 8, and 14 dpc, using a commercial ELISA kit (IDEXX Laboratories, Liebefeld, Switzerland). The blocking percentage values of samples were calculated from the OD at 450 nm following manufacturer’s instructions: values below 30% were considered negative, between 30 and 40% were considered doubtful, and above 40% were considered positive. The pigtype CSFV E^rns^ Ab test (Indical Bioscience GmbH, Leipzig, Germany) was used to detected antibodies against E^rns^ glycoprotein in serum samples following the manufacturer instructions. CSFV E^rns^-specific sample/positive (S/P) values ≥0.5 were considered positive, values between 0.3 and 0.5 as doubtful, and values < 0.3 as negative.

### 2.7 ELISA tests for IgG and IgA antibody isotypes against E2-CD154 chimeric protein

Specific IgG and IgA antibodies against the E2-CD154 chimeric protein were evaluated in serum and oral swabs at 14 and 28 dpv and at 0, 8, and 14 dpc in all groups from the efficacy study using an in-house ELISA method as previously described (Tarradas et al., 2012). Briefly, 0.6 µg/mL of E2-CD154 protein (IFA de Porvac^®^, lot. 13P.2318, CIGB) in sodium carbonate–bicarbonate buffer (0.05 M NaHCO_3_, 0.05 M Na_2_CO_3_, pH 9.4) was used to coat high-binding Costar 96 plates (Corning) overnight at 4°C. After washing three times (0.05% Tween in PBS), free active sites were blocked using a buffer containing 5% skim milk in 0.05% Tween in PBS during 1 h at 37°C. Serum samples were 1:100 prediluted in the same blocking buffer for IgG detection and 1:50 for IgA detection and incubated at 37°C for 2 h. After further washing, anti-swine IgG peroxidase conjugate (1:10,000; Sigma-Aldrich) or anti-swine IgA peroxidase conjugate (1:5,000, AbD Serotec, Oxford, UK) were diluted in blocking buffer, and plates were incubated at 37°C for 1 h. The antibodies were detected with the HRP substrate 3,3′,5,5′-tetramethylbenzidine (Calbiochem). Finally, the reaction was stopped with 1 M sulphuric acid, and the absorbance was measured at 450 nm. Results were expressed as optical density (OD).

### 2.8 Virus neutralization test

Serum samples were tested for neutralizing antibody titers against CSFV Margarita, Alfort/187, and Diepholz1/Han94 strains using a neutralization peroxidase-linked assay (NPLA) (Terpstra et al., 1984). The neutralization titers were expressed as the serum reciprocal dilution that neutralized 100 TCID_50_ of each specific viral strain in 50% of the culture replicates. As previously established, neutralizing antibody titers of 1:35 were considered to have protective capacity against CSFV (Terpstra and Wensvoort, 1988).

### 2.9 Determination of serum IFN-α levels by ELISA

The IFN-α concentration in serum was determined using a previously described in-house ELISA test (Diaz De Arce et al., 1992; Guzylack-Piriou et al., 2004). Anti-IFN-α monoclonal antibodies (K9 and K17) and serial dilutions of recombinant IFN-α protein (PBL Biomedical Laboratories, Piscataway, NJ, USA) were employed as a standard in this assay. Serum samples from pigs at 8 and 14 dpc were evaluated. The OD obtained from the different concentrations of the standards were used to perform a regression curve to quantify the concentration of IFN-α in serum samples, with the results being expressed as units/mL.

### 2.10 CSFV RNA detection and virus isolation

The tonsil samples collected after necropsy were ground in Eagle’s minimum essential medium, supplemented with 2% penicillin (10,000 U/mL) and streptomycin (10,000 U/mL) in a dilution 1:10 and centrifuged at 13,000 rpm for 10 min. Viral RNA was extracted from 200 μL of serum, oral and rectal swabs, and tonsil homogenate samples using the MagAttract 96 cador Pathogen Kit (Qiagen, Hilden, Germany), following the manufacturer’s instructions. The RNA was resuspended in a final volume of 100 μL and stored at −80°C until further use.

For CSFV RNA detection, the generic CSFV real-time reverse transcription-polymerase chain reaction (RT-PCR) assay was performed in a blinded manner (Hoffmann et al., 2005). Reactions were performed using the AgPath-ID™ One-Step RT-PCR Reagents (Applied Biosystems, Waltham, MA, USA). Samples were considered positive when the cycle threshold (Ct) values were equal to or less than 40 and negative when fluorescence was undetectable. Samples were categorized as high (Ct < 21), moderate (Ct = 21-30), and low (Ct > 30) viral RNA loads. Tonsil’s homogenate collected after necropsy was subjected to virus isolation. Briefly, SK-6 cells were seeded in a 96-well plate and incubated at 37°C and 5% CO_2_. After 24 h, 100 μL of a 1:10 dilution of the tonsil homogenate from each sample was added to the cell culture. After 72 h of incubation at 37°C and 5% CO_2_, viral presence was assessed using the PLA assay (Wensvoort et al., 1986; Wang et al., 2020).

### 2.11 Statistical analysis

Differences among groups were assessed using the non-parametric Kruskal–Wallis test, followed by Dunn’s multiple comparisons post-test. *p* values < 0.05 were considered statistically significant. GraphPad Prism (version 8.3.0.) (www.graphpad.com) software was used for statistical analysis.

## 3 results

### 3.1 Optimized dose and inoculation frequency for maximum efficacy

**Figure 3:**
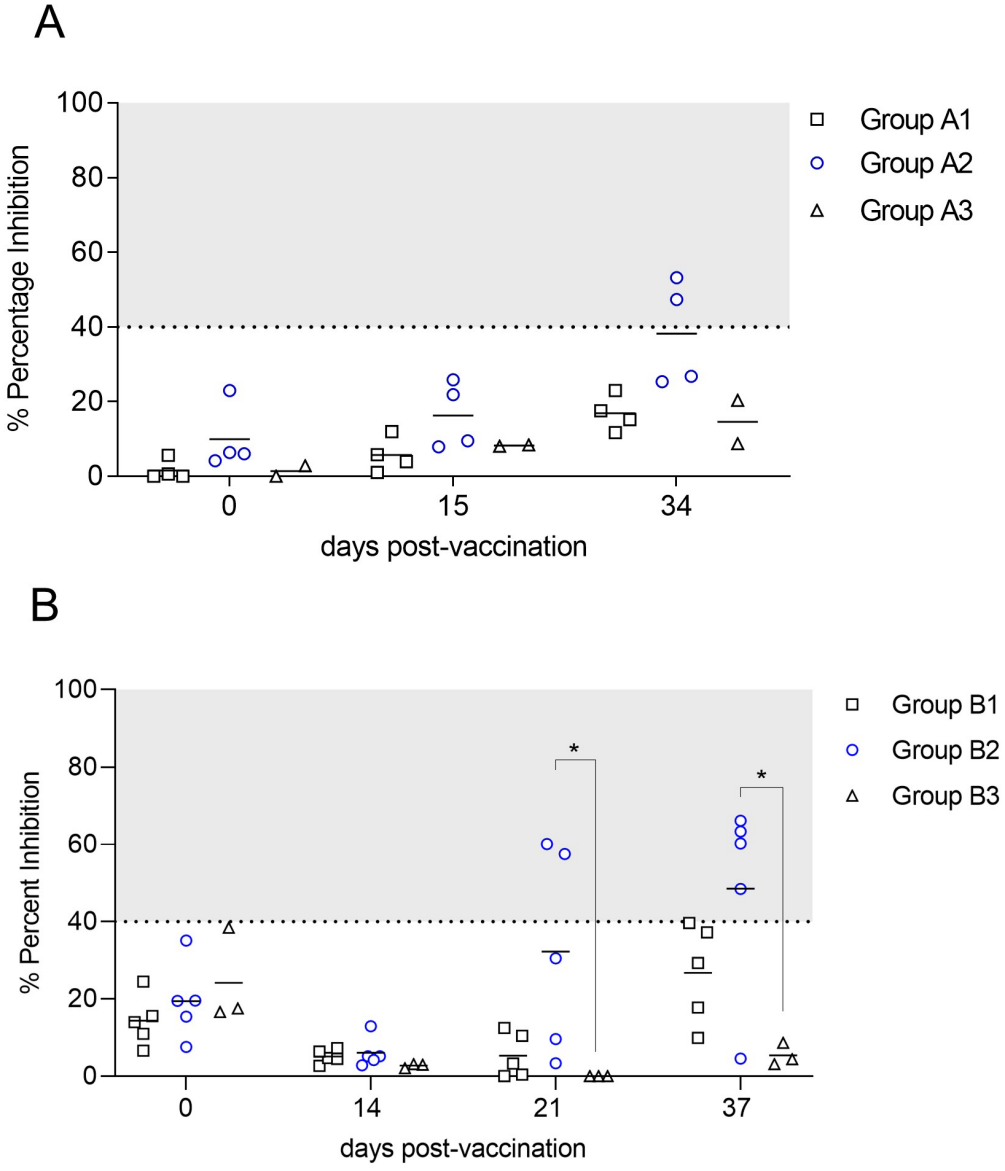
Anti-E2 antibody response in the dose and schedule immunization experiments. **(A)** Dose/effect experiment: group A1, two doses of 100 µg of E2-CD154 with 50 µg of c-di-AMP and another two of 100 µg of E2-CD154 with 200 µg of c-di-AMP; group A2, two doses of 500 µg of E2-CD154 with 50 µg of c-di-AMP and another two of 500 µg of E2-CD154 with 400 µg of c-di-AMP; group A3, PBS as negative control. **(B)** Number of immunizations experiment: group B1, three inoculations of 500 µg of E2-CD154 with 500 µg of c-di-AMP; group B2, nine inoculations of 500 µg of E2-CD154 with 500 µg of c-di-AMP; group B3, nine inoculations of PBS as negative control. Percent inhibition values ≥40% are considered positive. The Kruskal–Wallis test, followed by Dunn’s multiple comparison post-test, was performed to compare between groups. *indicates *p*-value < 0.05.

Two groups of domestic pig were inoculated with different doses of E2-CD154 protein and c-di-AMP. Pigs from group A2, which were immunized with the highest concentration of E2-CD154 and c-di-AMP, developed serum anti-CSFV E2 antibodies at the end of trial A (34 dpv), with a PI higher than 40% in two out of four pigs. Lower doses of E2-CD154 and c-di-AMP were not enough to develop anti-CSFV E2 antibodies in serum samples from group A1 (**Figure 3A**).

When optimizing the number of E2-CD154 sublingual inoculations in combination with c-di-AMP, pigs in group B2, which consisted of nine immunizations of 500 µg of E2-CD154 + 500 µg of c-di-AMP per dose, showed detectable anti-E2 antibodies in serum samples, with two out of five pigs reaching PI values >40% at 21 dpv. This response was statistically significant compared with group B3 (*p*-value < 0.05), administered with PBS. At 37 dpv, four out of five pigs in group B2 developed anti-E2 antibodies, showing a statistically significant difference compared with group B3 (*p*-value < 0.05). In contrast, no anti-CSFV E2 antibodies were detected in group B1, which received the same dose of E2-CD154 and c-di-AMP but in three immunization schedules (**Figure 3B**). Therefore, nine sublingual inoculations of 500 µg of E2-CD154 together with 500 µg of c-di-AMP were the dose and immunization schedule determined for the immunogenicity and efficacy study against CSFV.

### 3.2 E2-CD154 administered with c-di-AMP prevents clinical disease in pigs challenged with a highly virulent CSFV strain

**Figure 4:**
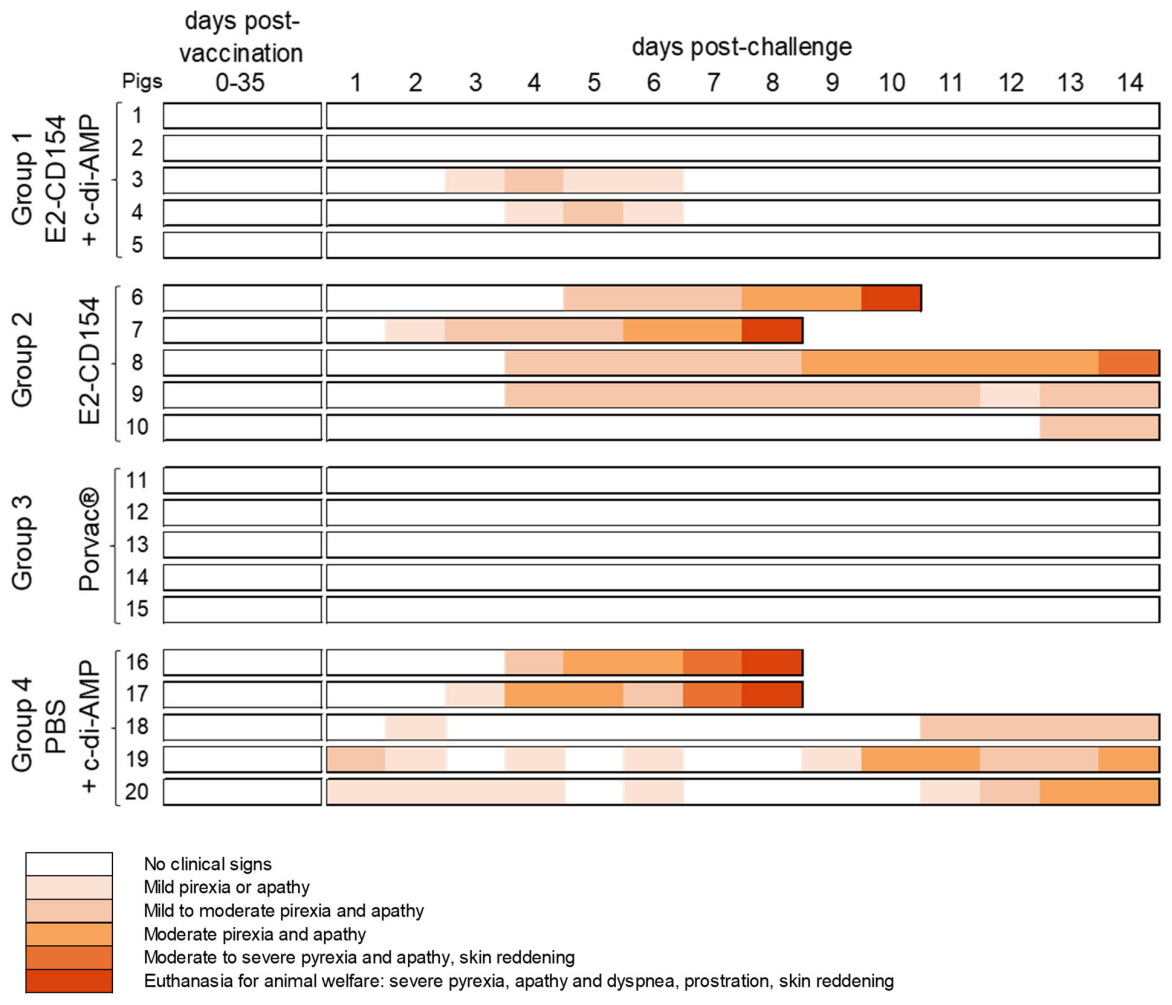
Clinical signs evaluated in pigs after immunization with E2-CD154 together with c-di-AMP (group 1), with E2-CD154 (group 2), with Porvac^®^ vaccine (group 3) or with PBS and c-di-AMP (group 4) and after CSFV Margarita strain challenge. The clinical signs were monitored daily during the trial. Different colors represent the severity of the clinical signs according to the legend.

After CSFV challenge, pigs immunized with E2-CD154 and c-di-AMP (group 1) were protected from the progressive CSF development during the 14 days that the challenge phase lasted. Only two out of five pigs showed transient mild to moderate pyrexia and apathy at 4 dpc (pig 3) and 5 dpc (pig 4). No further clinical signs were recorded in any pig from this experimental group until the end of the study (**Figure 4**). The absence of pathological findings after the necropsy supports the clinical protection observed in these animals.

However, in pigs administered with E2-CD154 alone, four out of five animals showed mild to moderate pyrexia and apathy from day 2 until 7 dpc. At 8 and 10 dpc, pigs 7 and 6 respectively showed severe pyrexia, dyspnea, conjunctivitis, cyanosis, skin reddening, tremors, and prostration and were euthanized for ethical reasons. The other three pigs in this group reached the end of the study (14 dpc) showing mild to moderate (pigs 9 and 10) and moderate to severe (pig 8) CSF clinical sings (**Figure 4**). Petechial hemorrhages were observed in kidney, spleen, stomach, and intestine tissue mainly in animal numbers 6, 7, and 8. Notably, total absence of clinical signs and pathological findings after necropsy was observed in pigs from group 3, vaccinated intramuscularly with Porvac^®^ vaccine, after CSFV challenge (**Figure 4**). By contrast, in animals from group 4 (PBS + c-di-AMP), the clinical signs were found shortly after the CSFV challenge. Two pigs were euthanized for ethical reasons at 8 dpc (pigs 16 and 17) and the other three reached the end of the study with mild to moderate (pig 18) and with moderate (pigs 19 and 20) CSF clinical signs (**Figure 4**). Petechial hemorrhages in kidney, spleen, stomach, and intestine tissue were observed in all the animals from the group, which are all typical pathological findings observed during CSF.

### 3.3 Combined administration of E2-CD154 and c-di-AMP limits CSFV detection after viral challenge

**Figure 5:**
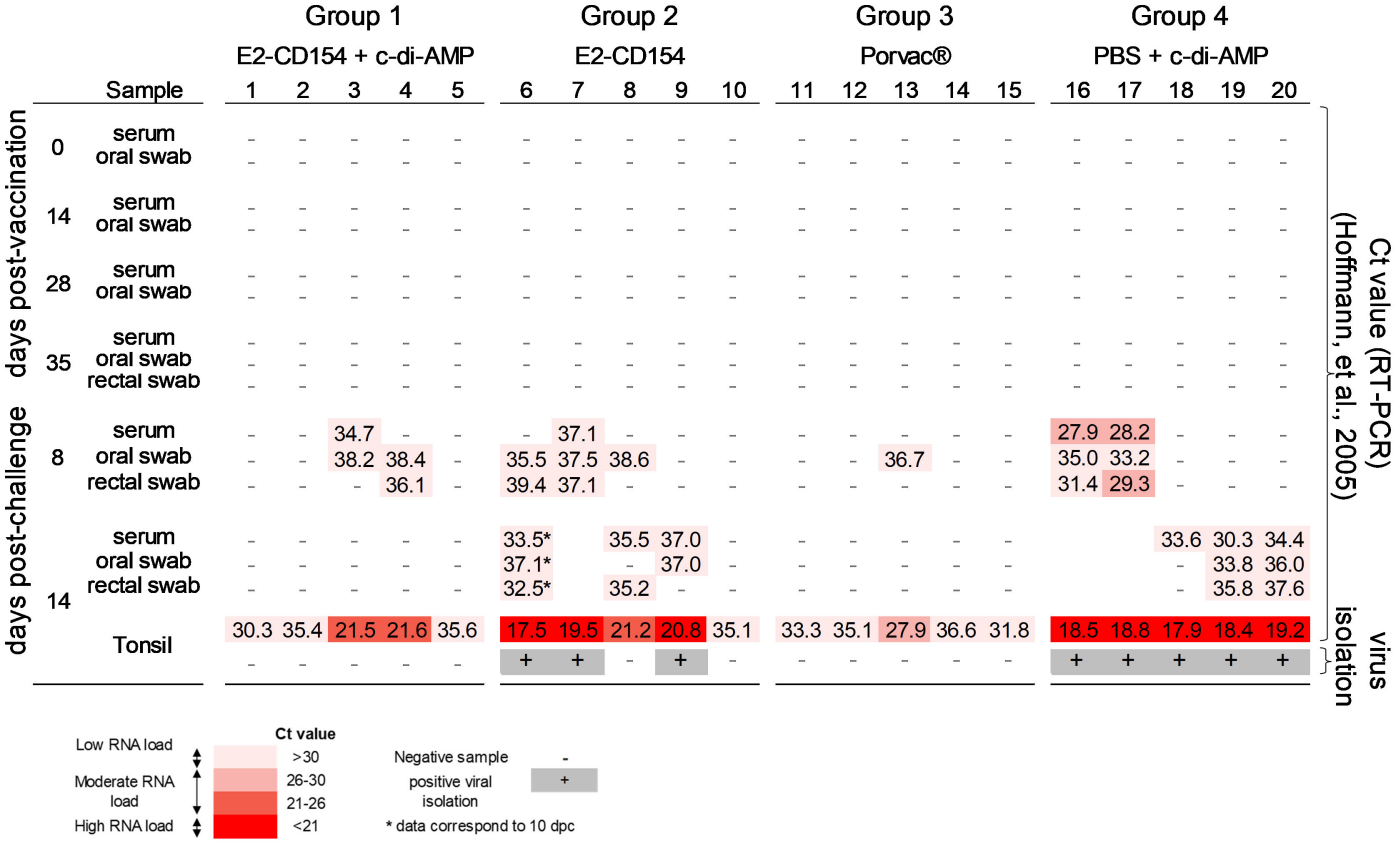
CSFV RNA detection in serum, oral, and rectal swab and tonsil samples at 0, 14, 28, and 35 days post-vaccination (dpv) and at 8 and 14 days post-challenge (dpc), using RT-PCR test (Hoffmann et al., 2005). The RNA load is represented as low (Ct > 30), moderate (Ct= 21-30), or high (Ct < 21) in different red colour intensity according to the Ct value. Ct values above 40 were considered negative. CSFV isolation from tonsils were determined by PLA, at the end of the study or when pigs were euthanized, and positive results were represented by the (+) symbol.

After CSFV challenge, all animals from the control group (group 4), administered with PBS + c-di-AMP, were positive for CSFV RNA in clinical samples during the study. At 8 dpc, CSFV RNA was detected in two of five pigs with Ct values ranging from low to moderate RNA loads: in serum (27.9) and oral (35.0) and rectal (31.4) swabs from pig 16, and in serum (28.2) and oral (33.2) and rectal (29.3) swabs from pig 17. Subsequently at 14 dpc, viral RNA was detected in the three remaining pigs with Ct values correlating with low RNA loads (Ct values > 30): in the serum of pig 18, and in serum and oral and rectal swabs from pigs 19 and 20 (**Figure 5**). In this group, tonsil samples from all pigs were positive to CSFV RNA detection with high RNA load (Ct values ≤19.2). The molecular results in the tonsils were further confirmed using the CSFV isolation test, being all positive after the PLA assay (**Figure 5**).

In group 1, immunized with E2-CD154 + c-di-AMP, three out of five pigs were fully protected, testing negative for viral RNA in serum and swab samples during the study (pigs 1, 2, and 5). Only two pigs tested positive for CSFV RNA at 8 dpc, showing low RNA loads in serum (34.7) and oral swab (38.2) from pig 3 and in oral (38.4) and rectal (36.1) swabs from pig 4. All tonsil samples tested positive for CSFV RNA, with a moderate load in pigs 3 and 4 (Ct = 21.5 and 21.6) and a low RNA load in the remaining three pigs (Ct value >30). Interestingly, all tonsils were negative for virus isolation test (**Figure 5**).

In the case of group 2, immunized with E2-CD154 alone, CSFV RNA was detected in four out of five pigs in samples collected during the study, although with low RNA loads (Ct values >30). Pig number 10 was negative throughout the study in all samples analyzed. In tonsils, a high RNA load was detected in three out of five pigs (17.4, 19.5, and 20.8), whereas the other two animals showed a moderate (21.2) and low (>30) RNA load. Virus isolation test was positive in tonsil samples from pigs 6, 7, and 9, whereas CSFV isolation was negative in tonsil samples from pigs 8 and 10.

Finally, in pigs from group 3, vaccinated with Porvac^®^, CSFV RNA was detected in only one animal (pig 13), in an oral swab collected at 8 dpc, with a low Ct value (36.7). However, at the end of the study, all tonsil samples from this group tested positive for CSFV RNA detection: Four out of five pigs showed low RNA loads (>30), and one pig (pig 13) showed moderate RNA load (27.9). Nevertheless, virus isolation from the tonsils of all Porvac^®^-vaccinated pigs was negative (**Figure 5**).

### 3.4 Co-administration of E2-CD154 and c-di-AMP elicited an anti-CSFV E2 antibody response in serum

**Figure 6:**
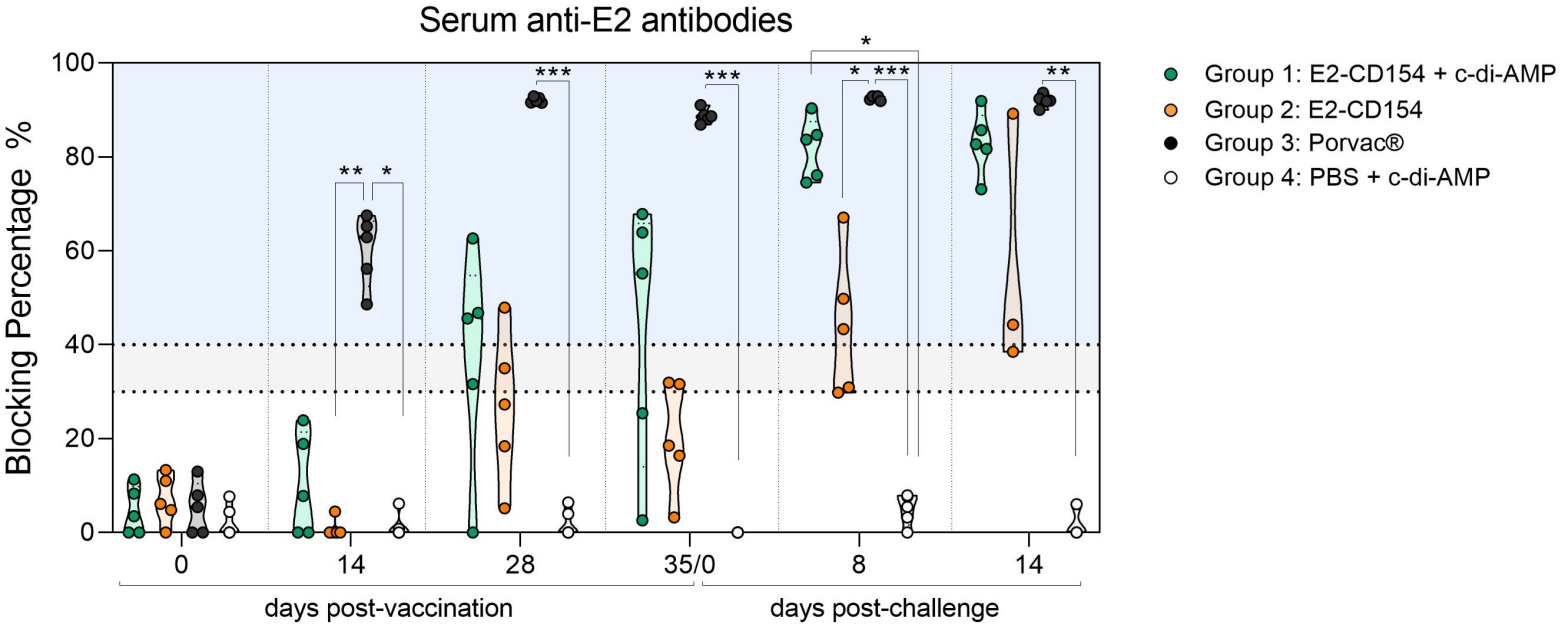
Antibody response against CSFV E2 protein generated in the sera of pigs after immunization and CSFV challenge. Anti-E2 antibodies were evaluated using a commercial ELISA test. The results are expressed in blocking percentage (%), and values above 40% (blue zone) were considered positive. Values between 30%-40% (grey zone) were considered as doubtful results. Group 1, immunized with E2-CD154 + c-di-AMP (green circles); group 2, immunized with E2-CD154 (orange circles); group 3, vaccinated with Porvac^®^ (black circles) and group 4, inoculated with PBS + c-di-AMP (white circles). All groups were challenged with CSFV Margarita strain. The Kruskal–Wallis test, followed by Dunn’s multiple comparison post-test, was performed to compare between groups. *, **, and *** indicate *p*-value < 0.05, 0.01, and 0.001, respectively.

**Figure 7:**
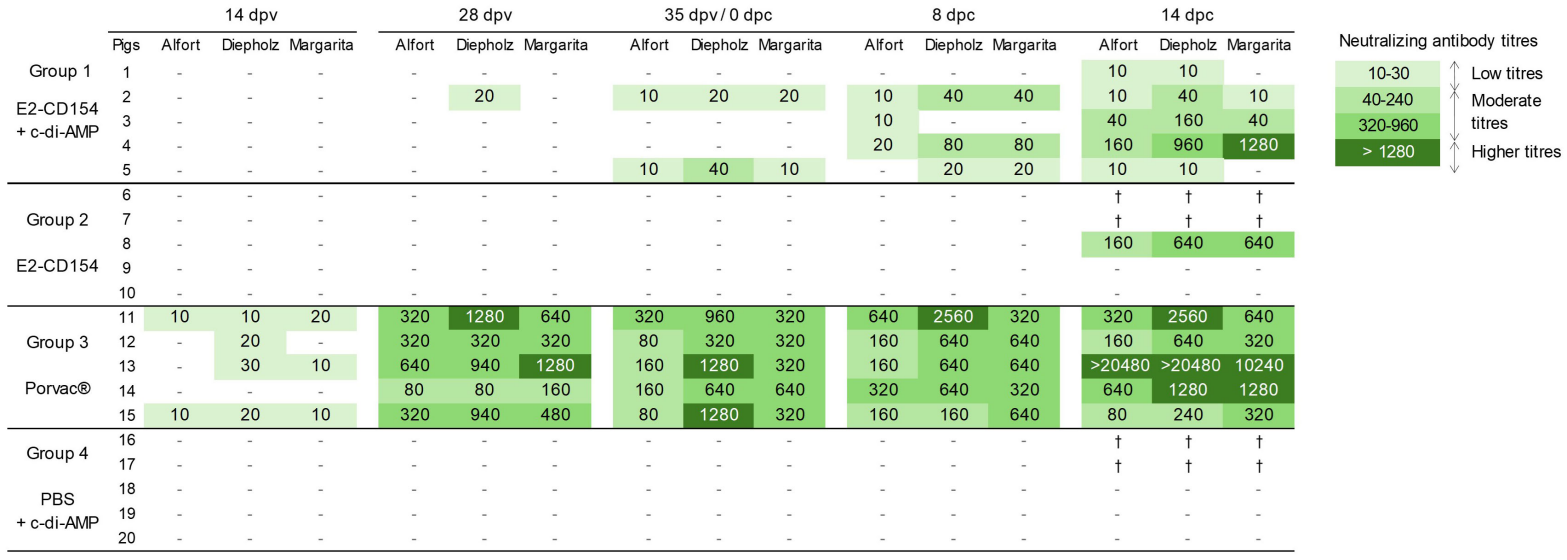
Kinetics of neutralizing antibody response after immunization and CSFV challenge. The neutralizing antibody titers against CSFV Alfort, Diepholz, and Margarita strains were determined by NPLA. Neutralizing antibody titers from low to high are represented on a scale from light to deep green. Negative samples are represented with a minus (–) symbol. A cross (†) indicates that the animal was euthanized.

No CSFV E2-specific antibodies were detected in animals from group 4 (inoculated with PBS + c-di-AMP) during the study (**Figure 6**). On the contrary, in pigs from group 1 (immunized with E2-CD154 + c-di-AMP), the CSFV E2-specific antibodies were first detected at 28 dpv in three out of five pigs whereas one pig showed a doubtful result and the other one was negative. At 35 dpv, three out of five pigs showed a blocking percentage between 50% and 70% in serum samples, whereas the other two showed a negative result. After CSFV challenge, all pigs from the group showed anti-CSFV E2 antibodies, and the blocking percentage increased to 70%-90% at both 8 and 14 dpc. These values at 8 dpc were significantly higher than those observed in group 4, which received c-di-AMP alone (*p*-value < 0.05) (**Figure 6**). The activation of this CSFV humoral response was further confirmed by a serum neutralization test, which showed low titers in the serum of one pig at 28 dpv and in the serum of two pigs at 35 dpv. After the CSFV challenge, four out of five pigs developed neutralizing antibodies with low to moderate titers at 8 dpc. By 14 dpc, all pigs in this group had neutralizing antibodies against at least two of the three CSFV strains tested. Two pigs exhibited low titers, whereas the remaining three showed moderate to high titers, reaching values up to 1:1,280 against the challenge strain (**Figure 7**).

On the other hand, in group 2 (immunized with E2-CD154 alone), anti-E2 antibodies were first detected at 28 dpv in one pig (48%). Another pig showed a doubtful value, whereas the remaining three pigs tested negative. At 35 dpv, two pigs showed doubtful values whereas the other three pigs were negative. After CSFV challenge, anti-E2 antibodies were detected at 8 dpc in three out of five pigs (with blocking percentage ranging from 40% to 70%), whereas the other two showed doubtful results. At 14 dpc, of the three remaining pigs, two showed anti-E2 antibody responses with blocking percentages of 90% and 44%, respectively, whereas the third pig showed a doubtful result (**Figure 6**). Despite this, the serum neutralization test detected no neutralizing antibodies in pigs from this group until the end of the study (14 dpc), when only one pig (pig 8) tested positive with moderate titers (1:640) against the challenge virus strain (**Figure 7**).

Finally, in pigs from group 3 (vaccinated with Porvac^®^), the CSFV-specific antibodies were detected in all serum samples as early as 14 dpv, with blocking percentages ranging from 50% to 70% (**Figure 6**) and being statistically significant with group 2 (*p*-value < 0.01) and group 4 (*p*-value <0.05). At this time, low titers of neutralizing antibodies were detected in four out of five pigs (**Figure 7**). At 28 dpv, the blocking percentages raised up to 90%-100% and continued that high until the end of the study, showing statistically significant differences compared with the control group (group 4) (*p*-value < 0.01-0.00.1) and with the group 2 at 8 dpc (*p*-value < 0.05) (**Figure 6**). This was consistent with the neutralization assay, which showed moderate to high titers of neutralizing antibodies against all three CSFV strains tested at 28 dpv, ranging from 1:80 to 1:1280. The titers in this group continued to increase until the end of the study (14 dpc) reaching values as high as 1:20,480 (**Figure 7**).

No CSFV E^rns^-specific antibodies were detected in any pig following E2-CD154 vaccination and CSFV challenge. Only one animal in group 2 (pig 8), immunized with E2-CD154 alone, showed a doubtful result (S/P= 0.42) at 8 dpc (data not shown).

### 3.5 Combined administration of E2-CD154 and c-di-AMP elicits systemic IgG and IgA responses against CSFV

**Figure 8:**
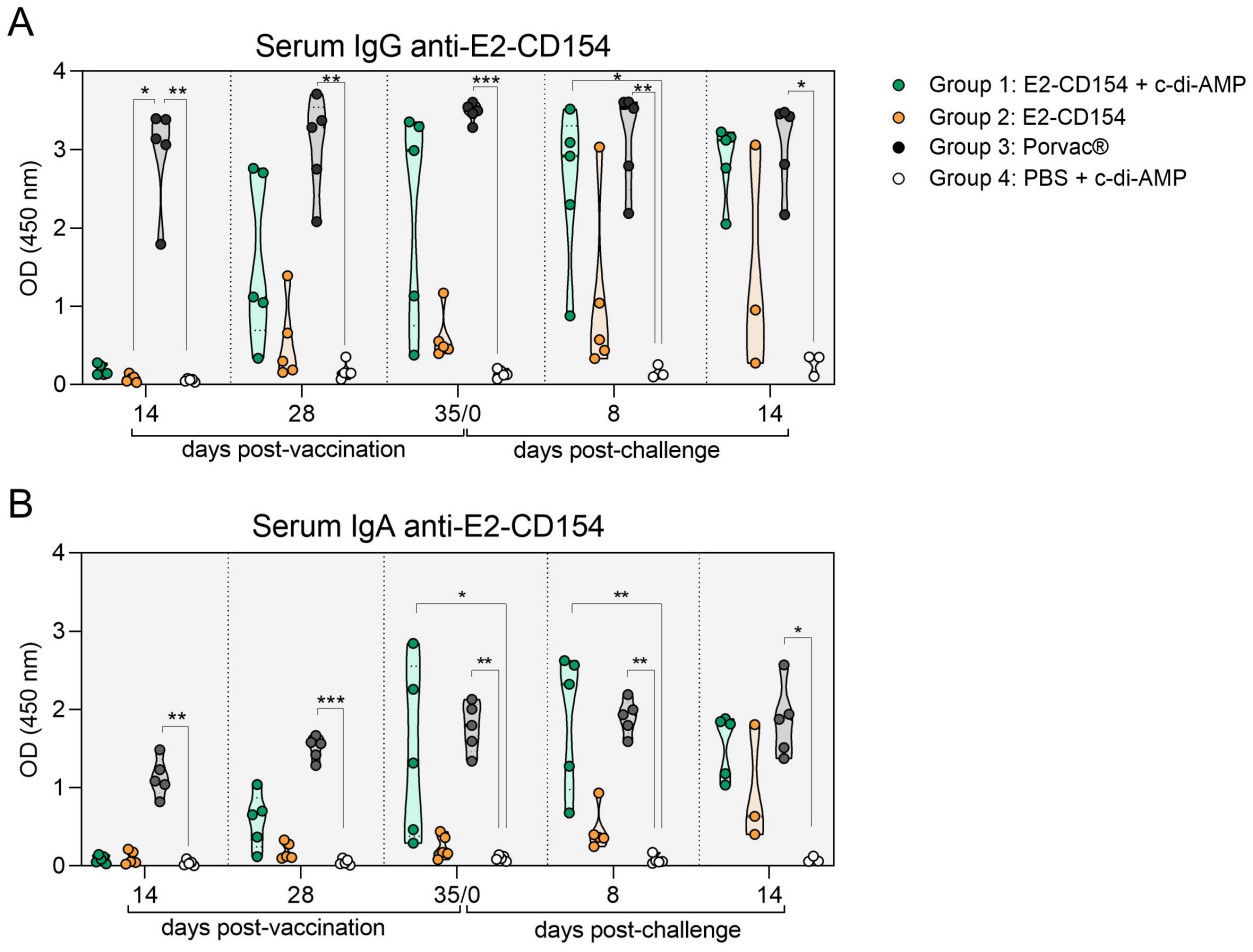
Anti-E2-CD154 antibody response generated in pigs after immunization and CSFV challenge in serum samples. **(A)** IgG antibodies and **(B)** IgA antibodies against E2-CD154 chimeric protein in serum samples. The results were expressed in OD. Group 1, pigs immunized with E2-CD154 + c-di-AMP (green circles); group 2, immunized with E2-CD154 (orange circles); group 3, pigs vaccinated with Porvac^®^ vaccine (black circles) and group 4, pigs inoculated with PBS + c-di-AMP (white circles). All groups were challenged with CSFV Margarita strain. The Kruskal–Wallis test, followed by Dunn’s multiple comparison post-test, was performed to compare between groups. *, **, and *** indicate p-value < 0.05, 0.01, and 0.001, respectively.

The IgG antibody response against the E2-CD154 chimeric protein was first detected at 14 dpv in group 3 (vaccinated with Porvac^®^), when all pigs exhibited increased IgG levels (one animal with OD = 1.7 and the remaining four with OD >3), showing statistically significant differences compared with group 2 (*p*-value < 0.05) and with the control group (*p*-value < 0.01). IgG values in this group remained high throughout the study, with OD consistently above 2 and statistically higher than those of the control group at all the time points (*p*-values = 0.05-0.001). In group 1 (immunized with E2-CD154 and c-di-AMP), IgG antibodies were first detected at 28 dpv, when two pigs reached OD = 2.7. At 35 dpv, three animals showed IgG levels with OD values >2 and by 8 dpc, this response was observed in four pigs with values significantly higher than those of the control group (*p*-value < 0.05). By the end of the study (14 dpc), all pigs in this group had OD values above 2, reaching levels comparable with those recorded in the Porvac^®^-vaccinated group. In contrast, in group 2 (immunized with E2-CD154 alone), IgG levels remained below OD = 1 throughout the study, except for one animal that showed OD = 3 at 8 and 14 dpc. No specific IgG responses were detected in group 4 (control) (**Figure 8A**).

The IgA antibody response followed a similar pattern to that of IgG. In group 3 (Porvac^®^), IgA was first detected at 14 dpv, with OD values ranging from 0.8 to 1.5 and increased progressively to 1.2-2.6 by the end of the study. These values were statistically higher than those observed in the control group (*p*-value = 0.05-0.001). In group 1 (E2-CD154 plus c-di-AMP), IgA responses appeared at 28 dpv in three pigs with OD values >0.7. By 35 dpv, three animals reached OD values between 1.3 and 2.9, and after CSFV challenge (8 dpc), three pigs exceeded an OD = 2.3, whereas the remaining two stayed below OD = 1.5, with values statistically significant compared with the control group at 35 dpv (*p*-value < 0.05) and 8 dpc (*p*-value < 0.01). At 14 dpc, all pigs in this group showed IgA responses with OD values between 1 and 2. On the other hand, in group 2 (E2-CD154 alone), IgA values remained below 0.5 during vaccination, with a modest increase in one pig at 8 dpc (OD = 1) and another animal reaching OD = 1.8 at 14 dpc. No IgA was detected in group 4 (control) (**Figure 8B**). In addition, no IgG or IgA antibodies were detected in oral swabs from any group (data not shown).

### 3.6 The E2-CD154 administrated with c-di-AMP protects pigs from an excessive IFN-α response following viral challenge

**Figure 9:**
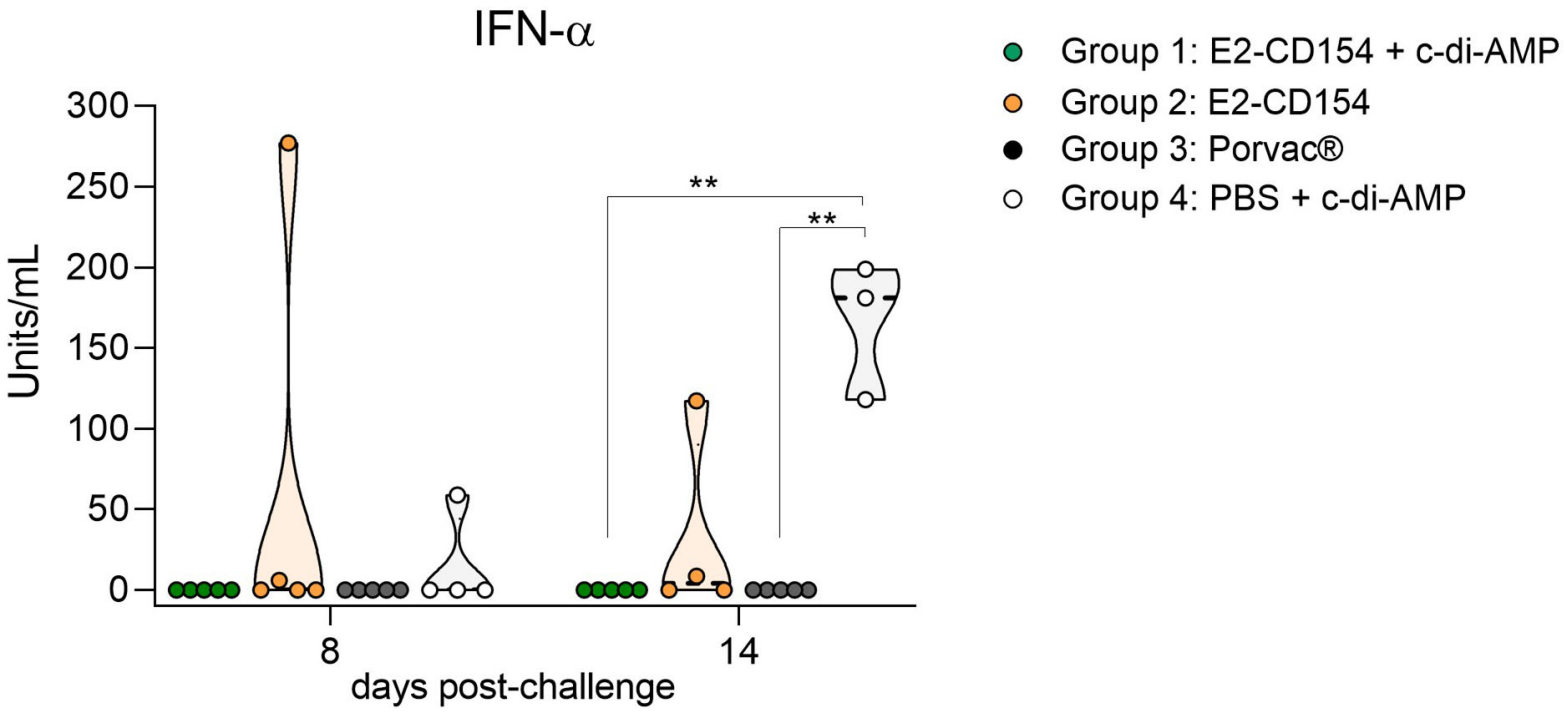
IFN-α response in the sera from pigs after 8- and 14-days post-challenge with CSFV. The results were expressed in units/mL. Group 1, pigs immunized with E2-CD154 + c-di-AMP (green circles); group 2, immunized with E2-CD154 (orange circles); group 3, pigs vaccinated with Porvac^®^ vaccine (black circles) and group 4, pigs inoculated with PBS + c-di-AMP (white circles). One data point in group 2 at 14 days post-challenge, corresponds to an animal that was euthanized and analyzed at 10 days post challenge. The Kruskal–Wallis test, followed by Dunn’s multiple comparison post-test, was performed to compare between groups. **indicates p-value < 0.01.

The evaluation of IFN-α response after CSFV challenge revealed the absence of IFN-α in the serum of pigs from group 1 (immunized with E2-CD254 + c-di-AMP) and group 3 (vaccinated with Porvac^®^). However, one pig in group 2 (immunized with E2-CD154) showed a high serum concentration of IFN-α at 8 dpc (277 units/mL) and at 14 dpc (117 units/mL). In group 4, control group, one out of four pigs showed increased levels of IFN-α at 8 dpc (59 units/mL). At the end of the study (14 dpc), all three remaining pigs showed increased levels of IFN-α in serum samples, reaching concentrations of up to 200 units/mL. These values were significantly higher than those observed in groups 1 and 4 (*p*-value < 0.05) (**Figure 9**).

## 4 Discussion

Unlike injectable vaccines, mucosal delivery is non-invasive, which improves compliance in both human and veterinary medicine, and would facilitate the vaccination of wildlife (Kraan et al., 2014; Wang et al., 2015; Trincado et al., 2021). Adjuvants are key to shaping immune responses, influencing their strength, quality, and duration. Cyclic dinucleotides (CDNs), like c-di-AMP, are promising new adjuvants with strong immunostimulatory effects (Burdette et al., 2011). Preclinical studies have demonstrated that CDNs can induce robust innate immune responses and promote both cellular and humoral immunity, with c-di-AMP being particularly effective in enhancing mucosal responses against infectious diseases (Woodward et al., 2010; Ebensen et al., 2011; Cheng et al., 2022). Most studies have used a mouse model to assess the potential of c-di-AMP as a vaccine adjuvant. However, its efficacy against viral infections in large mammals such as pigs has not yet been thoroughly studied. To our knowledge, c-di-AMP has previously been evaluated in pigs as a vaccine prototype adjuvant against a bacterial pathogen, administered via the intramuscular and intranasal routes, although its efficacy upon pathogen challenge was not clearly demonstrated (Bettin et al., 2025).

In this study, CSFV was used as a model to evaluate the potential of c-di-AMP as an adjuvant for a subunit vaccine prototype based on the E2-CD154 chimeric protein, co-administered via the sublingual mucosal route in domestic pigs. No side effects were observed during the immunization period, indicating that c-di-AMP was well tolerated by pigs. Following CSFV challenge, our data showed that the co-administration of E2-CD154 with c-di-AMP conferred superior protection compared with administration of the E2-CD154 protein alone. Three out of five animals receiving the E2-CD154 and c-di-AMP combination exhibited a complete absence of clinical signs. Although the other two developed mild symptoms only for 2 days, they fully recovered, and all pigs in the group completed the study without clinical manifestations. In terms of clinical signs, these outcomes were comparable with those observed with the intramuscular Porvac^®^ vaccine, used as a positive control, in line with the well-established protective efficacy of this subunit vaccine against CSF (Sordo-Puga et al., 2021).

The immunogenic E2 glycoprotein of CSFV linked to porcine CD154, as a novel molecular adjuvant, constitute the basis of the Porvac^®^ vaccine. CD154 is a natural CD40 ligand, expressed on activated CD4^+^ T cells, that activates antigen-presenting cells (APCs), thereby enhancing antigen presentation, co-stimulatory signals, and cytokine production to promote strong adaptive immunity (Elgueta et al., 2009). CSF vaccination strategies depend on the disease’s epidemiology, target population, domestic pigs and/or wild boar, and economic context (Ganges et al., 2020). Given the high transmissibility of CSFV and the diverse clinical manifestations that can coexist, vaccines developed against CSF must not only adhere to the DIVA concept but also demonstrate high efficacy in preventing viral replication and spread (Ganges et al., 2020; Coronado et al., 2021). In this regard, c-di-AMP administration together with E2-CD154 protein hindered CSFV replication. Only two pigs immunized with c-di-AMP and E2-CD154, which exhibited mild and transient clinical signs, tested positive for low levels of CSFV RNA in serum, rectal, and oral swabs. However, no CSFV RNA was detected in any sample at the end of the study, in line with the absence of clinical signs. A similar pattern was observed in the group vaccinated with Porvac^®^, thereby reinforcing the efficacy of both immunization strategies. Therefore, the adjuvant properties of c-di-AMP, the primary focus of this investigation, significantly contribute to the induction of robust virological protection in swine.

Tonsils are the target organs for CSFV replication and from where CSFV subsequently spreads to lymphoid tissues (Ressang, 1973; Belák et al., 2008; Blome et al., 2017). CSFV RNA was consistently detected in the tonsils of all animals, yet the RNA loads differed markedly among experimental groups. High viral RNA levels were observed in all control pigs and in several animals immunized with E2-CD154 alone. In contrast, the remaining pigs in the E2-CD154 group and those receiving either E2-CD154 combined with c-di-AMP or Porvac^®^ vaccine exhibited low to moderate RNA loads in tonsillar tissue. Strikingly, infectious viruses could only be recovered from the tonsils of control animals and from the subset of E2-CD154-immunized pigs with high CSFV RNA loads. Conversely, no viable CSFV was isolated from pigs with low to moderate RNA loads in tonsil samples, specifically two pigs immunized with E2-CD154 alone, those co-administered with E2-CD154 and c-di-AMP, and those immunized with Porvac^®^. These findings indicate that while the E2-CD154 recombinant protein partially limited viral replication in tonsillar tissue, the inclusion of c-di-AMP as an adjuvant substantially enhanced protective efficacy, resulting in complete abrogation of detectable infectious virus. Similarly, Porvac^®^ vaccination conferred robust protection, preventing viral persistence. It is important to note the high sensitivity of the RT-PCR assay (Hoffmann et al., 2005), which can yield positive results even in the absence of replicating virus. This may explain the detection of CSFV RNA in some immunized pigs, even though infectious virus could not be isolated in certain cases. These results indicate that immunization with the E2-CD154 antigen, administered either mucosal (adjuvanted with c-di-AMP) or intramuscularly (Porvac^®^ vaccine), does not entirely prevent CSFV from reaching the tonsils but effectively restricts replication and systemic spread. The presence of viral RNA in tonsils of protected animals therefore most likely reflects local viral persistence in the form of residual, non-infectious genomes rather than ongoing replication. This agreed with the absence of viral replication in serum and oral and rectal swabs, along with the lack of clinical signs in these groups. In addition, the CSFV-specific neutralizing antibody titers observed after viral challenge may play a key role in limiting viral replication and dissemination beyond the tonsils, not only in pigs vaccinated with Porvac^®^ and those immunized with E2-CD154 and c-di-AMP but also in the only pig immunized with E2-CD154 that developed neutralizing antibodies.

Vaccination with Porvac^®^ elicited high neutralizing antibody titers, consistent with previous findings (Sordo-Puga et al., 2025b), and correlated with strong protection against CSFV. Co-administration of E2-CD154 with c-di-AMP also induced a CSFV E2-specific antibody response, which was boosted post-challenge to levels comparable with those elicited by Porvac^®^ at 14 dpc. It should be noted that the limited number of animals, particularly at 14 dpc when euthanasia resulted in smaller effective group sizes, may have reduced the statistical power of some analyses. Nonetheless, the consistent trends observed across animals support the robustness of the results, as all pigs immunized with the Porvac^®^ vaccine or with E2-CD154 combined with c-di-AMP developed detectable antibodies, in contrast to the control group. This suggests that c-di-AMP enhances B-cell activation when combined with E2-CD154, as similar responses were not observed with E2-CD154 alone. Although antibodies from the group immunized with E2-CD154 and c-di-AMP exhibited lower neutralizing activity than those from Porvac^®^-vaccinated pigs, similar protection was achieved, implying a role for additional mechanisms—such as T-cell-mediated responses—in line with previous reports on protection mechanisms against CSFV (Ganges et al., 2005). Indeed, c-di-AMP has been shown to stimulate both humoral and cellular immunity upon infectious challenge (Sanchez et al., 2014; Ebensen et al., 2017; Schulze et al., 2017b; Ning et al., 2021). C-di-AMP likely enhances protective immunity through activation of the STING signaling pathway, leading to type I interferon induction, proinflammatory cytokine release, and activation of antigen-presenting cells that improve T-cell priming (Cheng et al., 2022). These innate mechanisms may contribute to the stronger humoral responses and protection observed in mucosal c-di-AMP–adjuvanted animals. Although direct assessment of cellular immunity (e.g., IFN-γ ELISPOT or cytokine profiling) was not performed, such analyses will be valuable in future studies to elucidate the T-cell–mediated components of the immune response. In addition, the absence of anti-E^rns^ antibodies throughout the study showed the DIVA capability of the E2-CD154 immunization strategy, as the antibody response was specific to E2 in all animals that showed humoral response. Control animals that received c-di-AMP alone failed to develop detectable antibodies, highlighting the severity of disease in the non-vaccinated, virus-challenged group. Acute CSF is characterized by profound immunosuppression, which in this study necessitated the euthanasia of affected pigs due to the rapid progression and severity of infection. The combination of shortened survival time and the marked immunosuppressive state associated with acute CSF precluded the development of measurable antibody responses against either of the analyzed glycoproteins, E2 or E^rns^ (Ganges et al., 2020).

Recent studies have shown that intramuscular and intranasal immunization using c-di-AMP as adjuvant induces a strong humoral IgG response in mice and pigs (Sanchez et al., 2014; Bettin et al., 2025). Here, pigs sublingually immunized with E2-CD154 and c-di-AMP showed an increased E2-CD154-specific IgG response after CSFV challenge, reaching levels comparable with those induced by Porvac^®^. Notably, c-di-AMP has elicited higher antigen-specific IgG levels than other adjuvants in protein-based immunizations (Ebensen et al., 2011; Volckmar et al., 2019). The systemic IgA antibody response against E2-CD154 protein after immunization and CSFV challenge mirrored the kinetics of IgG, which is consistent with previous reports of synchronous E2-specific IgG and IgA induction post-vaccination or infection (Panyasing et al., 2018). All pigs immunized with E2-CD154 and c-di-AMP developed systemic IgA after challenge, reaching levels like those vaccinated with Porvac^®^. Class switching to IgA can occur in both systemic and mucosal tissues, and serum IgA may reflect strong immune activation—either due to adjuvants like c-di-AMP or Montanide (used in Porvac^®^) or the migration of IgA-producing B cells from mucosal sites. Although secretory IgA was undetectable in mucosa in our study, systemic IgA may still signal mucosal immune involvement. Systemic IgA induction has previously been reported following mucosal immunization in human (Eijgenraam et al., 2008), and particularly with c-di-AMP as an adjuvant in mice (Ebensen et al., 2017). Notably, mucosal immunization with E2-CD154 alone induced weak IgA responses, underscoring c-di-AMP’s adjuvant potential in swine. The strong humoral response induced by E2-CD154 with c-di-AMP and by Porvac^®^ is reflected in the absence of serum IFN-α, which typically increases during CSFV infection and correlates with disease severity (Summerfield and Ruggli, 2015; Wang et al., 2022) but is absent after effective vaccination (Coronado et al., 2024). In contrast, pigs immunized with E2-CD154 alone but especially control pigs showed detectable IFN-α levels in sera, indicating ongoing viral replication and innate immune activation.

Taking together, this study reports, for the first time, the use of the STING agonist c-di-AMP as a mucosal adjuvant for a recombinant protein vaccine against a viral challenge in a large mammalian host, the pig. Our results demonstrate that sublingual mucosal delivery of the E2-CD154 chimeric protein formulated with c-di-AMP provided strong protection in domestic pigs, inducing humoral responses, effectively restricting CSFV replication and preventing CSF disease, comparable with the intramuscular injection of commercial Porvac^®^ vaccine. These findings represent an important step toward the development of a non-replicating mucosal CSF DIVA vaccine. Although the current dosing schedule successfully demonstrated the protective potential of the c-di-AMP adjuvanted formulation, follow-up studies are underway to optimize immunization regimens in pigs. These efforts aim to reduce the number of doses and explore oral delivery strategies, such as improving mucosal retention or implementing prime-boost regimens for practical field application. More broadly, this proof-of-concept approach advances the field of vaccinology and may provide a framework for the rational design of next-generation subunit vaccines targeting diverse viral pathogens across multiple mammalian species, including humans.

## Data Availability

The original contributions presented in the study are included in the article/supplementary material. Further inquiries can be directed to the corresponding author.
